# Substance Use, Suicidal Behavior, and Other Mental Health Outcomes Among Youth With Intersectional Sexual and Gender Diverse Identities

**DOI:** 10.1016/j.jaacop.2024.09.011

**Published:** 2025-01-09

**Authors:** Gladys N. Pachas, Harrison T. Reeder, A. Eden Evins, Kelly Casottana, Marta Borrego Mahiques, Alec Bodolay, Caroline A. Gray, Kevin W. Potter, Alex S. Keuroghlian, Randi M. Schuster

**Affiliations:** a Massachusetts General Hospital, Boston, Massachusetts; b Harvard Medical School, Boston, Massachusetts; c Fenway Institute, Boston, Massachusetts

**Keywords:** mental health, LGBTQIA2S+, disparities, suicide, depression

## Abstract

**Objective:**

Sexual diverse (SD) and gender diverse (GD) youth are at increased risk for adverse mental health outcomes compared to heterosexual/cisgender youth (HC). In this study, we aimed to examine youth risk profiles. We hypothesized that there would be a synergistic effect of SD+GD on mental health outcomes.

**Method:**

In 2022, a total of 23,915 adolescents in grades 6 to 12 from 60 schools completed the voluntary, confidential Substance Use and Risk Factor (SURF) survey. Questions included screeners for substance use and mental health distress. Regression models examined associations between mental health outcomes and HC, GD, SD, and SD+GD.

**Results:**

Mean age was 14.7 years; 25% identified as SD, 5.3% as GD, and 5.2% as SD+GD. Compared to HC, SD+GD youth had higher odds of depression (95% CI = 4.8-6.3), anxiety (95% CI = 4.9-6.5), suicidal thoughts (95% CI = 7.7-10.1), plans (95% CI = 6.7-8.8), attempts (95% CI = 7.0-10.7), nonsuicidal behavior (95% CI = 7.19-9.55), psychotic-like experiences (PEs) (95% CI = 4.1-5.4), emotional reactivity (ER) (95% CI = 22.1-24.5), alcohol use (95% CI = 1.2-2.4), cannabis use (95% CI = 2.3-4.0), smoking (95% CI = 9.4-26.6), and vaped nicotine (95% CI = 1.5-2.6) (all *p* < .04). Compared to SD only, SD+GD had higher odds of depression (95% CI = 1.5-2.0), anxiety (95% CI = 1.5-2.0), suicidal thoughts (95% CI = 1.7-2.3), plans (95% CI = 1.5-2.1), attempts (95% CI = 1.4-2.2), nonsuicidal behavior (95% CI = 1.67–2.27), PEs (95% CI = 1.6-2.2), ER (95% CI = 8.0-18.3), and smoking (95% CI = 2.3-7.8) (all *p* < .001). Compared to GD only, SD+GD had higher odds of depression (95% CI = 1.3-4.5), anxiety (95% CI = 1.8-6.4), suicidal thoughts (95% CI = 1.4-4.7), plans (95% CI = 1.1-4.1), nonsuicidal behavior (95% CI = 1.4-6.3), PEs (95% CI = 1.1-3.7), and ER (95% CI = 6.9-9.6) (all *p* < .03).

**Conclusion:**

Although the GD only group is small, these findings on intersectional SD+GD identities have significant implications for adolescent mental health.

## Plain language summary

This study examined mental health and substance use risks in adolescents who identify as sexually diverse (SD) or gender diverse (GD), and especially those who identify as both. The authors used the Substance Use and Risk Factor survey, and data were collected from nearly 24,000 adolescents from 60 schools in Massachusetts. The authors found that adolescents who identify as both (SD+GD) are at a significantly higher risk of experiencing depression, anxiety, suicidal thoughts and behaviors, and substance use compared to their heterosexual/cisgender peers. SD and GD adolescents are also at a higher risk of depression, anxiety, and emotional reactivity compared to heterosexual/cisgender peers. These findings suggest that the combined challenges of being both SD and GD put adolescents at a higher risk for adverse mental health outcomes, highlighting the need for specialized mental health support for this group.

The increasing prevalence of mental health problems among adolescents is a major public health concern. Rates of depression, anxiety, suicide, and drug overdose deaths have been steadily rising in adolescents since the late 2000s.^1^, ^2^, ^3^, ^4^ The 2021 Youth Risk Behavior Survey (YRBS) revealed that 42% of high school students reported persistent feelings of sadness or hopelessness in the previous year, 1 in 5 had thoughts of suicide, and 1 in 5 attempted suicide. Similarly, 23% of high school students reported currently using alcohol, 18% nicotine vapes, 16% marijuana, 4% smoking cigarettes, and 6% non-prescribed opioids.^5^

Previous studies have demonstrated disparities in mental health outcomes across groups of adolescents,^6^ notably including those who identify either as sexual diverse (SD) or gender diverse (GD).^7^ The Youth Risk Behavior Survey found significant differences between heterosexual youth and youth who identified as lesbian, gay, bisexual, queer, (LGBQ+). For example, 52% of LGBQ+ youth reported experiencing poor mental health in the past 30 days, in contrast to 22% of their heterosexual peers. Moreover, in the past year, 15% of heterosexual youth have seriously considered attempting suicide and 12% have made a suicide plan; for LGBQ+ youth, these figures increase to 45% and 37%, respectively.^5^

Historically, most research has focused on examining mental health outcomes of the LGBTQIA2S+ group as a single unit, collapsing across sexual and gender identities. Using this aggregate term to broadly refer to various identities not only assumes that all identities are similar, but also may disregard the uniqueness of experiences and the struggles associated with each identity.^8^

More recently, research has focused on assessing sexual orientation and gender identity within the LGBTQIA2S+ group as their separate identities. This separation by sexual diverse vs gender diverse identities allows for a better understanding of these groups’ individual needs, enabling targeted interventions and advancing clinical practice. These studies found that, compared to heterosexual cisgender (HC) youth, adolescents who identified as either SD or GD had higher odds of life dissatisfaction, poor self-esteem, depressive symptoms, suicidal thoughts and behaviors^9^, ^10^, ^11^, ^12^ and substance use.^11^^,^^13^^,^^14^ This increased negative mental health outcomes may be associated with the unique challenges and stressors that SD and GD adolescents encounter. Structural forms of social stigma and discrimination frequently experienced by LGBTQIA2S+ youth drive psychological distress and increased odds of engaging in risky health-related behaviors.^15^^,^^16^ On the other hand, inclusive and affirming school policies,^17^ and access to gender-affirming medical care, not only improve GD youths’ mental health to a level comparable to that of their cisgender peers, but also improve preventive care (eg, screening for cervical cancer among transgender male individuals).^18^^,^^19^

Although the body of literature examining SD and GD as 2 separate identities is growing,^6^^,^^11^^,^^20^^,^^21^ there is recent evidence suggesting that these SD and GD identities intersect. A school-based survey in Pittsburgh, Pennsylvania, found that 55% of GD adolescents also identified as SD.^22^ Reisner *et al.*^23^ found, in a US national probability sample of transgender adults, that 82.4% had a sexual minority identity. Another study found that SD individuals who also identified as transgender tended to identify with multiple sexual orientation identities compared to cisgender SD individuals.^24^ These findings challenge the research approach of examining GD and SD identities as 2 separate, mutually exclusive entities, and suggest the need to understand the characteristics and risk profiles of these intersecting identities. Interestingly, the 2015 Healthy Kids Colorado Survey^25^ and the 2022 national US survey of SD and GD youth^26^ found disparities characterized by increased risk of nonsuicidal self-injury, suicide attempts, and reported depressive symptoms in co-occurring SD+GD adolescents compared to GD or SD youth.^25^, ^26^, ^27^ This prior research, although scarce, suggests a potential synergistic effect of SD and GD identities. It is important to examine whether these disparities also affect other important mental health outcomes such as anxiety, suicidal thoughts and behaviors, substance use, and other transdiagnostic indicators of psychiatric distress and risk. Understanding these disparities might help us to have a better representation of the behaviors, experiences, challenges and needs of adolescents with co-occurring SD+GD,^6^ allowing providers to create more personalized, tailored, inclusive and effective interventions.^28^ Tailored interventions may, in time, help providers build trust and rapport, increase patient satisfaction and engagement, and decrease the effect of risk factors such as discrimination, stigma, and health disparities.

In this study, our aim was to characterize the mental health profile of SD and GD identities and to compare mental health risk profiles among youth who identified as HC, SD only, GD only, and SD+GD. We hypothesized that there would be a synergistic effect of having intersecting SD and GD identities, such that, when compared with SD (primary comparison), SD+GD would have higher odds of reporting depressive or anxiety symptoms, suicidal thoughts and behaviors, as well as substance use, emotional reactivity, and psychotic-like experience.

## Method

### Participants and Recruitment

Students in grades 6 to 12 from 24 middle schools, 34 high schools, and 2 combined middle/high schools across Massachusetts completed the 2022-2023 Mass General Hospital Substance Use and Risk Factor survey (SURF) from September 2022 to February 2023.^29^^,^^30^ Most participating schools (84.5%) were located in urban areas, and 14.5% of schools were in rural areas.^31^ In addition, the majority of schools (97%) had a Gay Straight Alliance or Genders and Sexualities Alliances (GSA) program in their school or in a school located in the same city or town. Study procedures were approved by the Massachusetts General Brigham Institutional Review Board.

### Data Collection

The SURF survey is a brief, confidential school-wide survey collected via electronic REDCap links through an opt-out parental consent process. Participating schools sent opt out letters to all parents/legal guardians of 6th through 12th grade students following schools’ preferred method of communication (through reverse 911 calls, e-mails, mass mailings) in the parent/legal guardian’s preferred language (English and 16 additional languages). The opt out letter indicated that school staff would be distributing a brief de-identified questionnaire, during a time selected by the school, to all students who were not withdrawn by their parents/legal guardians. All students who were not opted out by their parents/legal guardians were eligible to participate.

Participation in the survey was voluntary, and students could choose to add their contact information if they were interested in being contacted about research opportunities. Most completers participated anonymously; only 2.2% of the students included their contact information. No compensation was provided for survey completion. Questions in the survey included demographics, substance use, and psychiatric symptoms including current depressive and anxiety symptoms, past-year suicidal thoughts, and lifetime psychotic experiences. The survey was offered in English and 16 additional languages (Spanish, Portuguese, French, Arabic, Filipino, Ukrainian, Dari, Farsi, Haitian Creole, Bengali, Gujarati, Turkish, Amharic, Russian, and Simplified Chinese).

### Measures

#### Sexual and Gender Identities

Sexual orientation and gender identity were independently queried according to best practices guidelines.^32^ To characterize SD identity in this study, students answered the question “Do you think of yourself as: (1) straight or heterosexual, (2) gay or lesbian, (3) bisexual, (4) queer, (5) pansexual, (6) asexual, (7) something else, (8) questioning or still figuring it out, (9) I haven't thought about it or I don't know what this question means, and (10) I don't want to say.” Students were able to select 1 response only. Responses were dichotomized as straight or heterosexual (response option 1) vs SD (response options 2-7). Responses 8 to 10 were recoded as missing data.

To characterize GD identity in this study, students answered the question “What is your current gender identity? Please choose the option that best describes you: (1) boy/man/male, (2) girl/woman/female, (3) transgender boy/man/male, (4) transgender girl/woman/ female, (5) nonbinary, genderqueer, or not exclusively male or female, (6) another gender, (7) not sure, and (8) I don't want to say.” Students were able to select 1 response only. Responses were dichotomized as cisgender (responses 1 and 2) and GD (responses 3-6). Responses 7 and 8 were recoded as missing data.

### Outcome Assessments

Current (past 2 weeks) symptoms of depression and anxiety were assessed with the Four-Item Patient Health Questionnaire (PHQ-4),^33^ a validated screening tool able to identify adolescents with symptoms of depression and/or anxiety.^34^ with sum scores ≥3 for the first 2 items (GAD-2) suggestive of anxiety (range, 0-6) and sum scores ≥3 for the last 2 items (PHQ-2) suggestive of depression (range, 0-6).

Self-injurious thoughts and behaviors (past year), including suicidal thoughts, suicidal plan, suicidal attempt, and nonsuicidal self-harm, were assessed with 4 yes/no questions: (1) Did you ever have thoughts about killing yourself (ending your life)? (2) Did you think about how you would kill yourself? (3) Did you try to kill yourself? and (4) Did you hurt yourself on purpose without trying to kill yourself?”

Lifetime psychotic-like experiences (PEs) were assessed with the Adolescent Psychotic-like Symptom Screener (APSS),^35^ a 7-item self-report that cover 7 domains: visual and auditory hallucinations, paranoia, grandiosity, delusions of control, mind reading, and delusions of reference. Responses ranged from (0) no, never, (1) maybe, to (2) yes, definitely. We used scores of 2 or more as a cut off for being “at-risk” for PEs. These PEs are subsyndromal “psychotic-like” symptoms that are reported by approximately 22% of adolescents.^36^ PEs represent a transdiagnostic marker for an increased risk for psychiatric disorders, with up to a 10-fold risk for developing a primary psychotic disorder, a 4-fold risk for mood disorders, and a 3-fold risk for anxiety disorders.^37^^,^^38^

The Emotional Reactivity Scale (ERS)^39^ assessed for emotion sensitivity, arousal or intensity, and persistence. It is a 21-item self-report; total sum scores, ranging from 0 to 84, were used in this study. Higher scores were suggestive of greater emotional reactivity. Data has shown that high levels of emotional reactivity predicted a range of psychiatric disorders at follow-up. The most consistent associations emerged for major depression subsyndromal panic attacks.^40^

Use of alcohol, cannabis, smoked tobacco, and vaped nicotine were collected for 2 time points: lifetime (ever used/tried) and past 30 days use (none, once, less than once a week, at least once a week, and daily). Past-month substance use frequency was recoded as less than once a week vs at least weekly use.

### Statistical Analyses

Records were excluded if the student incorrectly answered 2 embedded attention check questions, “Please choose response D for this question. This is just to see if you are paying attention.” Records were also excluded if the student completed less than 60% of the unbranched survey questions.

Demographic characteristics and outcomes under study are summarized by HC, GD only, SD only, and SD+GD, using counts and proportions for categorical variables, and means and standard deviations for continuous variables.

For each binary mental health outcome, we considered a logistic regression model using a categorical variable representing HC, GD only, SD only, and SD+GD identities. First, we reported the associations between each outcome and youth identifying as SD only or GD only, relative to HC identity. Unadjusted odds ratios are reported, as well as odds ratios adjusted for age and sex. Finally, we reported unadjusted and adjusted odds ratios for the intersectional SD+GD identity vs HC, GD only, and SD only. This highlighted the impact on mental health outcomes of intersectional identity relative to SD identity alone or GD identity alone and HC. We used a similar regression approach for the continuous outcome of ERS total score, considering a linear regression model.

A Benjamini–Hochberg^41^ procedure was used to control for multiple comparisons. Statistical analyses were performed using Stata/BE 17.0

## Results

### Sample Characteristics

A total of 1,213 students (4.8% of total enrollment) were opted out by their parents or legal guardians. The 2022 SURF survey had a mean completion rate of 76.1% (standard deviation, ±0.4%). In all, 23,915 adolescents completed the SURF survey and were included in the analysis. Participants excluded from analyses because of predefined quality control parameters (failure of 2 or more attention check and/or <60% completion) had 44% of missing data for demographics. We found that these participants tended to be older (14.9 ± 2.1 years vs 14.7 ± 1.9 years), male (29.4% vs 27.3%), non-Hispanic (44.4% vs 16.9%), and were in high school (65.7% vs 34.3%) (all *p* < .001).

Participant characteristics by sexual and gender identity are shown in Table 1. Mean age was 14.8 years (±1.9), 6,578 (33%) were in middle school, 16,011 (66%) were in high school, 10,949 (51.4%) were female (sex at birth), and 4,934 (20.9%) were Hispanic. Regarding sexual and gender identity, 17,571 (79.2%) identified as HC (45.4% female gender), 53 (0.3%) as GD only, (0.2% transgender, 0.1% nonbinary/another gender, 8.5% I don’t know/didn’t want to say), 2,730 (12.8%) as SD only (11.7% gay/lesbian, 0.6% bisexual/other, 6.2% didn’t know/didn’t want to say), and 1,023 (4.8%) as SD+GD. (Table S1, available online). Aside from a higher proportion of SD students in high schools (middle school: 8% vs high school: 13.1%; *p* < .001), the proportion of students identifying as HC, GD, and SD+GD was comparable in middle school (HC 71.8%, GD only 0.3%, SD only 8.0%, SD+GD 4%) vs high school: HC 74.4%, GD only 0.2%, SD only 13.1%, SD+GD 4.4% (all *p* values nonsignificant) (Table S2, available online).

**Table 1:** Demographics and Clinical Characteristics by Identity

Demographics	Identity			
	HC	GD Only	SD Only	SD+GD
	n = 17,571 (82.2%)	n = 53 (0.3%)	n = 2,730 (12.8%)	n = 1,023 (4.8%)
Age, y, mean (SD)	14.74 (±1.9)	14.94 (± 2.4)	15.19 (± 1.9)	14.82 (± 2.0)
Sex				
Female	7,987 (45.6)	25 (47.2)	2,137 (78.4)	800 (79.1)
Male	9,533 (54.4)	28 (52.8%)	588 (21.6)	212 (20.9)
Race				
Asian	1,018 (5.9)	4 (7.7)	135 (5.0)	64 (6.3)
Haitian/African American	1,047 (6.0)	2 (3.8)	155 (5.7)	39 (3.9)
Multiple	1,351 (7.8)	15 (28.8)	263 (9.7)	139 (13.7)
Other^a^	1,744 (10.1)	5 (9.6)	240 (8.9)	78 (7.7)
White	12,188 (70.3)	26 (50.0)	1,909 (70.7)	691 (68.3)
Grade				
Middle school	5,614 (32.0)	22 (41.5)	629 (23.1)	313 (30.7)
High school	11,909 (68.0)	31 (58.5)	2,093 (76.9)	706 (69.3)
Ethnicity				
Hispanic	3,538 (20.3)	19 (36.5)	579 (21.4)	223 (21.9)
Non-Hispanic	13,863 (79.7)	33 (63.5)	2,124 (78.6)	793 (78.1)
**Clinical Characteristics**				
Clinical outcomes				
Anxiety symptoms (% ≥3 on PHQ4-A)	3,610 (20.7)	16 (30.2)	1,445 (53.4)	664 (65.4)
Depression symptoms (% ≥3 on PHQ4-D)	2,566 (14.7)	15 (28.3)	1,075 (39.7)	534 (52.6)
Psychotic-like experiences (% ≥2 on APSS)	2,576 (14.9)	15 (28.3)	761 (28.5)	445 (44.4)
Suicidal thoughts and behaviors (past year)				
Thoughts	1,934 (11.1)	16 (30.2)	1,056 (39.0)	562 (55.5)
Plan	1,459 (8.3)	13 (24.5)	816 (30.1)	436 (43.0)
Attempt	300 (1.7)	5 (9.4)	239 (8.8)	150 (14.8)
Nonsuicidal self-harm (past year)	1,213 (7.0)	9 (17.0)	742 (27.4)	434 (42.8)
Substance use past 30 days (at least once/wk)				
Alcohol	396 (2.3)	5 (9.4)	80 (2.9)	41 (4.0)
Cannabis	479 (2.7)	5 (9.4)	191 (7.0)	72 (7.1)
Smoking tobacco	44 (0.3)	5 (9.4)	19 (0.7)	30 (3.0)
Vaped nicotine	534 (3.1)	3 (5.7)	188 (6.9)	67 (6.6)
ERS: mean total score (SD)	16.49 (± 18.0)	26.96 (± 22.8)	35.04 (± 22.0)	43.76 (± 23.1)

Note: Data are n (%) unless otherwise noted. The PHQ-D and the PHQ-A show the percentage of adolescents who scored ≥3 on each scale. APSS shows the percentage of adolescents who scored ≥2 on the scale. APSS = Adolescent Psychotic-like Symptom Screener; ERS = Emotional Reactivity Scale total score; GD = gender diverse; HC = hetero/cisgender; PHQ-A = Patient Health Questionnaire—Anxiety subscale; PHQ-D = Patient Health Questionnaire—Depression subscale; SD = sexual diverse, SD+GD = intersectional sexual diverse and gender diverse identities.

a Other race = Hawaiian + American Indian + Middle Eastern.

### Associations With Clinical Outcomes (Adjusted by Age and Sex)

#### GD Only vs HC

**Figure 1:**
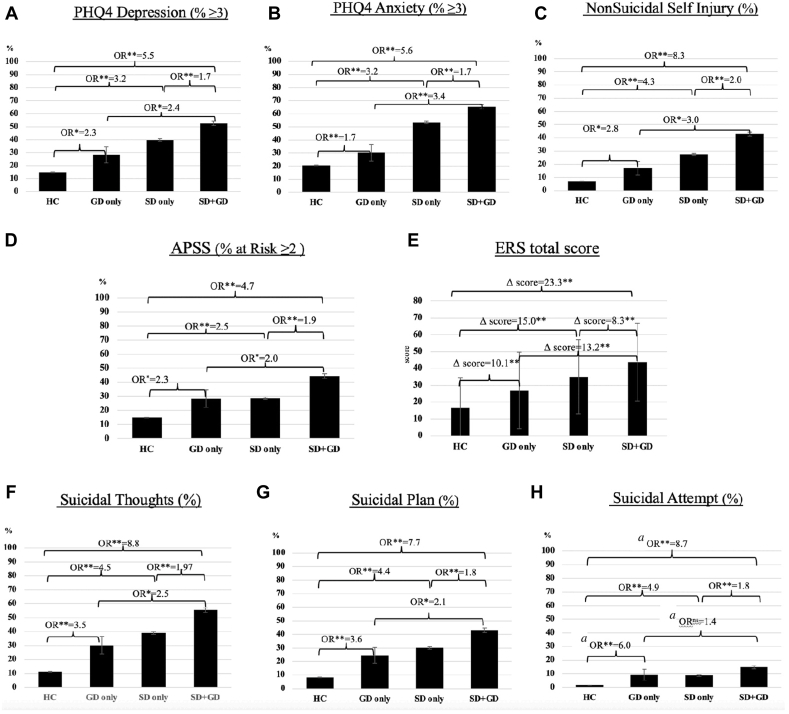
Effect of Sexual Diverse (SD) Only, Gender Diverse (GD) Only, and SD+GD on Clinical Outcomes ***Note:****(a) Adjusted odds ratios (OR) showing a compounding effect of SD+GD on depressive symptoms (PHQ-D ≥3) compared to single identities. (b) Adjusted OR showing a compounding effect of SD+GD on anxiety symptoms (PHQ-A ≥3) compared to single identities. (c) Adjusted OR showing a compounding effect of SD+GD on nonsuicidal self-injury compared to single identities. (d) Adjusted OR showing a compounding effect of SD+GD on psychotic-like symptoms (APSS ≥2) compared to single identities. (e) Difference in Emotional Reactivity Scale scores in SD, GD, and SD+GD adolescents. (f) Adjusted OR showing a compounding effect of SD+GD on self-report of suicidal thoughts compared to single identities. (g) Adjusted OR showing a compounding effect of SD+GD on self-report of having a suicidal plan compared to single identities. (h) Adjusted OR showing a compounding effect of SD+GD on nonsuicidal self-injury compared to single identities. APSS = Adolescent Psychotic-like Symptom Screener; ERS = Emotional Reactivity Scale total score; HC = hetero/cisgender; ns = non-significant; PHQ-A = Patient Health Questionnaire—Anxiety subscale; PHQ-D = Patient Health Questionnaire—Depression subscale.* ^*a*^*Because of the small sample size, comparisons including the GD group should be considered as suggestive findings for suicidal attempts.* ∗p < .*05;* ∗∗p < .*001*.

Compared to HC, GD only youth had higher odds of reporting depressive symptoms (odds ratio [OR] = 2.3, 95% CI = 1.2-4.1, *p* = .008), of being in the at-risk category for PEs (OR = 2.3, 95% CI = 1.3-4.3, *p* = .006), as well as past-year suicidal thoughts (OR = 3.5, 95% CI = 2.0-6.3, *p* < .001), suicidal plan (OR = 3.6, 95% CI = 1.9-6.7, *p* < .001), suicidal attempt (OR = 6.0, 95% CI = 2.4-15.1, *p* < .001), and nonsuicidal self-injurious behavior (OR = 2.8, 95% CI = 1.3-5.5, *p* = .006). GD only youth had significantly higher emotional reactivity scores than HC youth (Δ score = 10.1, 95% CI = 5.0-15.2, *p* < .001). Association with anxiety was non-significant (*p* = .110) (Table 2, Figure 1)

**Table 2:** Single and Synergistic Effect of Sexual and Gender Identities on Mental Health Outcomes (Adjusted by Sex and Age)

Measure	OR (95% CI)				
	GD only vs HC	SD only vs HC	SD + GD vs HC	SD + GD vs GD	SD + GD vs SD
Clinical outcomes					
Depression symptoms (PHQ4-D ≥3)	**2.3 (1.2-4.1)∗**	**3.2 (2.9-3.5)∗∗**	**5.5 (4.8-6.3)∗∗**	**2.4 (1.3-4.5)∗**	**1.7 (1.5-2.0)∗∗**
Anxiety symptoms (PHQ4-A ≥3)	**1.6 (0.9-3.0)∗∗**	**3.2 (3.0-3.5)∗∗**	**5.6 (4.9-6.5)∗∗**	**3.4 (1.8-6.4)∗∗**	**1.7 (1.5-2.0)∗∗**
Psychotic-like experiences (APSS ≥2)	**2.3 (1.3-4.3)∗**	**2.5 (2.2 – 2.7)∗∗**	**4.7 (4.1-5.4)∗∗**	**2.0 (1.1-3.7)∗**	**1.9 (1.6-2.2)∗∗**
Suicidal thoughts and behaviors (past year)					
Thoughts	**3.5 (2.0-6.3)∗∗**	**4.5 (4.1-4.9)∗∗**	**8.8 (7.7-10.1)∗∗**	**2.5 (1.4-4.7)∗**	**2.0 (1.7-2.3)∗∗**
Plan	**3.6 (1.9-6.7)∗∗**	**4.4 (4.0-4.8)∗∗**	**7.7 (6.7-8.8)∗∗**	**2.2 (1.1-4.1)∗**	**1.8 (1.5-2.1)∗∗**
Attempt	**6.0**^a^**(2.4-15.1)∗∗**	**4.9 (4.1-5.9)∗∗**	**8.7**^a^**(7.0-10.7)∗∗**	1.5^a^ (0.6-3.7)^ns^	**1.8**^a^**(1.4-2.2)∗∗**
Nonsuicidal self-harm	**2.8 (1.3-5.7)∗**	**4.3 (3.8-4.7)∗∗**	**8.3 (7.2-9.6)∗∗**	**3.0 (1.4-6.3)∗**	**2.0 (1.7-2.3)∗∗**
Substance use (past month)					
Alcohol (weekly use or more)	**3.4 (1.1-9.9)∗**	1.2 (0.9-1.5)^ns^	**1.7 (1.2-2.4)∗**	0.5 (0.2-1.6)^ns^	1.5 (1.0-2.2)^ns^
Cannabis (weekly use or more)	**2.9 (1.0-8.4)∗**	**2.8 (2.3-3.4)∗∗**	**3.1 (2.3-4.0)∗∗**	1.0 (0.4-3.1)^ns^	1.1 (0.8-1.5)^ns^
Smoking tobacco (weekly use or more)	**36.7**^a^**(12.5-107.6)∗∗**	**3.7 (2.1-6.5)∗∗**	**15.9**^a^**(9.4-26.6)∗∗**	0.4^a^ (0.1-1.3)^ns^	**4.3**^a^**(2.3-7.8)∗∗**
Vaped nicotine (weekly use or more)	1.4 (0.4-4.8)^ns^	**2.0 (1.6-.4)∗∗**	**1.97 (1.5-2.6)∗∗**	1.4 (0.4-5.0)^ns^	1.0 (0.8-1.4)^ns^

	Δ Score (95% CI)				
	GD only vs HC	SD only vs HC	SD + GD vs HC	SD + GD vs GD	SD + GD vs SD
ERS (total score)	**10.1 (5.0-15.2)∗∗**	**15.0 (14.2–15.8)∗∗**	**23.3 (22.1-24.5)∗∗**	**8.3 (6.9-9.6)∗∗**	**13.2 (8.0-18.3)∗∗**

Note: *p* Values have been corrected for multiple comparisons with Benjamini–Hochberg procedure. APSS = Adolescent Psychotic-like Symptom Screener; ERS = Emotional Reactivity Scale total score; GD = gender diverse; HC = hetero/cisgender; ns = non-significant; PHQ-A = Patient Health Questionnaire—Anxiety subscale; PHQ-D = Patient Health Questionnaire—Depression subscale; SD = sexual diverse; SD+GD = intersectional sexual diverse and gender diverse identities. Results that are considered statistically significant, based on a *p* value < 0.05, have been highlighted in bold text.

∗*p* < .05; ∗∗*p* < .001.

a Because of the small sample size, comparisons including the GD group should be considered as suggestive findings for smoking tobacco and suicidal attempts.

#### SD Only vs HC

Compared to HC, SD only youth had higher odds of reporting depressive symptoms (OR = 3.2, 95% CI = 2.9-3.5, *p* < .001), anxiety symptoms (OR = 3.2, 95% CI = 3.0-3.5, *p* < .001), PEs (OR = 2.5, 95% CI = 2.2-2.7, *p* < .001), as well as past-year suicidal thoughts (OR = 4.5, 95% CI = 4.1-4.9, *p* < .001), suicidal plan (OR = 4.4, 95% CI = 4.0-4.8, *p* < .001), suicidal attempt (OR = 4.9, 95% CI = 4.1-5.9, *p* < .001), and nonsuicidal self-injurious behavior (OR = 4.3, 95% CI = 3.8-4.7, *p* = .006). Similarly, GD only and SD only youth had significantly higher emotional reactivity scores than HC youth (Δ score = 15.0, 95% CI = 14.2-15.8, *p* < .001) (Table 2, Figure 1)

#### SD+GD vs HC

Compared to HC, youth who identified as SD+GD had 5.5 times higher odds of reporting depressive (95% CI = 4.8-6.3, *p* < .001) symptoms, 5.6 times higher odds of reporting anxiety symptoms (95% CI = 4.9-6.5, *p* < .001), 4.7 times higher odds of being in the at-risk category for PEs (95% CI = 4.1-5.4, *p* < .001), 8.8 times higher odds of having suicidal thoughts (95% CI = 7.7-10.1, *p* < .001), 7.7 times higher odds of having a suicide plan (95% CI = 6.7-8.8, *p* < .001), 8.7 times higher odds of having attempted suicide (95% CI = 7.0-10.7, *p* < .001), and 8.3 times higher odds of nonsuicidal self-injurious behavior (95% CI = 7.2 – 9.6, *p* < .001). SD+GD youth had higher emotional reactivity than HC youth (Δ score = 23.3, 95% CI = 22.1-24.5, *p* < .001) (Table 2, Figure 1).

#### SD+GD vs GD Only

Compared to GD only, SD+GD youth had 2.4 times higher odds of depressive symptoms (95% CI = 1.3-4.5, *p =* .005), 3.4 times higher odds of anxiety symptoms (95% CI = 1.8-6.4, *p* < .001), 2.0 times higher odds of being in the at-risk category for PEs (95% CI = 1.1-3.7, *p* = .030), 3.0 times higher odds of nonsuicidal self-injurious behavior (95% CI = 1.4 – 6.3, *p* = .004), 2.5 times higher odds of having suicidal thoughts (95% CI = 1.4-4.7, *p* = .002), and 2.2 times higher odds of having a suicide plan (95% CI = 1.1-4.1, *p* = .019). SD+GD youth had a higher emotional reactivity than HC (Δ score =13.2, 95% CI = 8.0-18.4, *p* < .001) (Table 2, Figure 1).

#### SD+GD vs SD Only (Primary Comparison)

Compared to SD only, youth who identified as SD+GD had 1.7 times higher odds of depressive symptoms (95% CI = 1.5-2.0, *p* < .001), 1.7 times higher odds of anxiety symptoms (95% CI = 1.5-2.0, *p* < .001), 1.9 times higher odds of being in the at-risk category for PEs (95% CI = 1.6-2.2, *p* < .001), 2.0 times higher odds of nonsuicidal self-injurious behavior (95% CI = 1.7-2.3, *p* < .001), 2.0 times higher odds of having suicidal thoughts (95% CI = 1.7-2.3, *p* < .001), 1.8 times higher odds of having a suicide plan (95% CI = 1.5-2.1), *p* < .001), and 1.8 times higher odds of having attempted suicide (95% CI = 1.4-2.2, *p* < .001). Intersectional SD+GD identities had higher emotional reactivity than HC (Δ score = 8.3, 95% CI = 6.9-9.6, *p* < .001) (Table 2, Figure 1).

Unadjusted associations of sexual and gender identities with clinical outcomes are reported Table S3, available online.

### Associations With at Least Weekly Current Substance Use (Adjusted by Age and Sex)

#### GD Only vs HC

**Figure 2:**
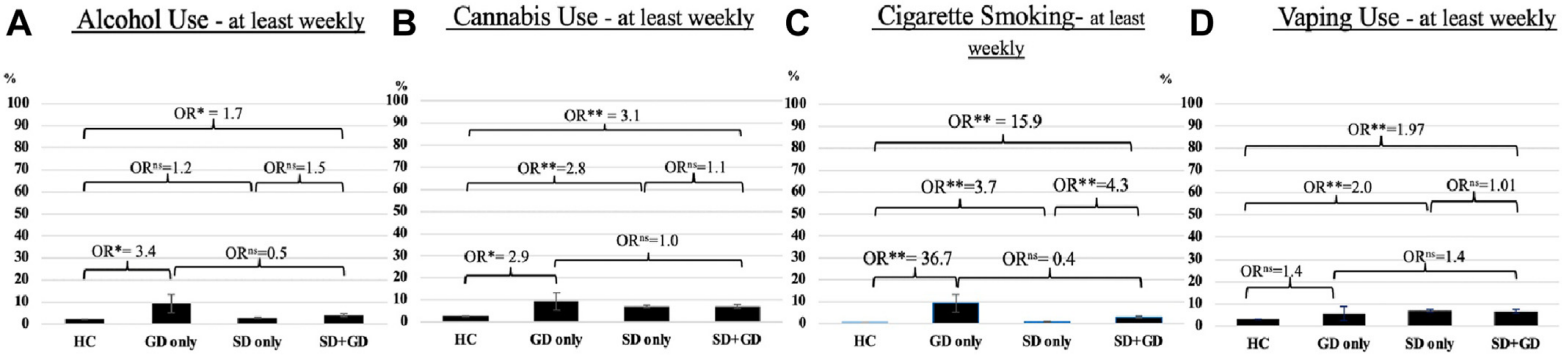
Sexual Diverse (SD) Only, Gender Diverse (GD) Only, and SD+GD Effect on Substance Use (Percentage of at Least Weekly Use) ***Note:****(a) Adjusted odds ratios (OR) showing a compounding effect of SD+GD on alcohol use compared to hetero/cisgender (HC) but not when compared to single identities. (b) Adjusted OR showing a compounding effect of SD+GD on cannabis compared to HC but not with single identities. (c) Adjusted OR showing a compounding effect of SD+GD on cigarette smoking compared to HC and SD only. (d) Adjusted OR showing a compounding effect of SD+GD on vaping nicotine compared to HC but not with single identities. *p* Values have been corrected for multiple comparisons. AllORwere adjusted for age and sex. ns = Nonsignificant.* ^*a*^*Because of the small sample size, comparisons including the GD group should be considered as suggestive findings for cigarette smoking.* ∗p <.*05;* ∗∗p <.*001.*

Compared to HC, GD only youth had higher odds of drinking alcohol (OR=3.4, 95% CI = 1.1-9.9, p=0.028), cannabis use (OR=2.9, 95% CI = 1.0-8.4, p=0.046), and cigarette smoking (OR = 36.7, 95% CI = 12.5-107.6, *p* < .001). Associations with nicotine vaping was non-significant (*p* = .601) (Table 2, Figure 2).

#### SD Only vs HC

Compared to HC, SD only youth had higher odds of cannabis use (OR = 2.8, 95% CI = 2.3-3.4, *p* < .001), cigarette smoking (OR = 3.7, 95% CI = 2.1-6.5, *p* < .001) and nicotine vaping (OR = 2.0, 95% CI = 1.6-2.4, *p* < .001). Associations with alcohol use were non-significant (*p* = .253) (Table 2, Figure 2).

#### SD+GD vs HC

Compared to HC, SD+GD youth had 1.7 times higher odds of drinking alcohol (95% CI = 1.2-2.4, *p* = .03), 3.1 times higher odds of using cannabis (95% CI = 2.3-4.0, *p* < .001), 2.0 times higher odds of vaping nicotine (95% CI = 1.5-2.6, *p* < .001), and 15.9 times higher odds of smoking tobacco (95% CI = 9.4-26.6, *p* < .001) (Table 2, Figure 2).

#### SD+GD vs GD Only

There were no differences between youth who identified as SD+GD vs GD only across any substance use outcome (all *p* > .14) (Table 2, Figure 2).

#### SD+GD vs SD Only (Primary Comparison)

Compared to SD only, youth who endorsed intersectional SD+GD identities had 4.3 times higher odds of smoking tobacco (95% CI = 2.3-1.3, *p* < .001) (Table 2, Figure 2). There was no association between SD only and use of alcohol, cannabis, or nicotine vaping (all *p* > .06) (Table 2, Figure 2).

Unadjusted associations of sexual and gender identities with at least weekly current substance use are reported in Table S3, available online.

### Sensitivity Analysis

A sensitivity analysis was performed including previously excluded undetermined answers (not sure, questioning, I don’t want to say) with 2 models: in model 1, an undetermined group was added to their corresponding SD or GD group; in model 2, an undetermined group was added to the HC group. We examined the effects of identities on mental health outcomes. In model 1, effect sizes and significance remained for all outcomes except alcohol and cannabis use (GD vs HC) (Table S4a, available online). In model 2, effect sizes and significance remained, except for depression and self-harm (SD+GD vs GD) (Table S4b, available online).

## Discussion

Our findings in a school-based sample of middle and high school students replicate prior reports that highlight disparities in mental health outcomes among SD or GD youth when compared to heterosexual or cisgender peers. These disparities are characterized by higher prevalence of substance use^42^ and increased risk for experiencing depressive and anxiety symptoms,^10^^,^^11^ self-injurious thoughts and behaviors,^9^^,^^12^ and other adverse mental health outcomes among LGBTQIA2S+ youth.^11^

Our results add to the growing evidence that SD and GD identities are likely not isolated from each other.^22^^,^^24^ Our results show, for example, that although most (95%) adolescents who identified as GD also identified as SD, around 30% of adolescents who identified as SD also identified as GD.

Moreover, our results support our hypothesis of a synergistic effect of having intersectional SD+GD identities, such that, when compared to SD only, SD+GD youth had higher odds of reporting depressive or anxiety symptoms, suicidal thoughts and behaviors, nonsuicidal self-harm, as well as tobacco smoking. There was also a synergistic effect when assessing for presence of lifetime PEs^43^^,^^44^ and levels of emotional reactivity.^45^ Although SD youth who endorse PEs are at higher risk for negative mental health outcomes such as depression, anxiety, and suicidal thoughts and behaviors,^43^ the presence of PEs may occur as part of normal development, especially in childhood and adolescence.^35^ For example, we found that 15% of middle school students and 13.6% of high school students endorsed at least 1 PE. Similarly, emotional reactivity has been associated with depressive symptoms and suicidal thoughts and behaviors in childhood.^45^ We found that intersectional SD+GD identities had higher odds of being at risk for lifetime PEs (2 or more PEs) and obtained an even higher level of emotional reactivity than all of the other groups (HC, GD only, and SD only youth). Importantly, these transdiagnostic and other indicators of psychiatric distress and risk may also be mediated by social adversity such as bullying, lack of social support, discrimination, and drug use.^44^^,^^45^

These results, in line with previous data,^25^ highlight these groups of adolescents with intersectional SD+GD identities as groups at higher risk within an already at-risk group. There is, however, a gap in understanding the risk and protective factors at this SD+GD intersection. According to the minority stress model, or in case of intersectionality, the multiple minority status, structural forms of social and identity-based stigma, and the discrimination frequently experienced by LGBTQIA2S+ youth increased psychological distress as well as the odds of engaging in health risk–related behaviors.^15^ This supports the need to examine this SD+GD identity not simply as an additive effect of SD and GD, but truly as intersectional identities that are “co-constructed and interdependent”,^46^ that the traumatic experiences and marginalization these individuals with intersectional identities face are not simply additive, they are interconnected.

Although mental health and substance use disparities exist across groups (SD vs HC and GD vs HC), we did not find a synergistic effect of having an intersectional identity (SD+GD) when compared to SD on alcohol, vaping, or cannabis use. Previous research on intersectional identities have proposed that this lack of synergistic effect might be associated with coping mechanisms and resources developed by these youth to cope with minority stressors associated with their SD or GD identity, developing stronger resilience to overcome the added effects of having intersectional identities against higher odds of substance use.^47^

There are several study limitations. First, this is a cross-sectional study, and therefore causation cannot be determined. Second, this study relied upon self-report measures only, often depending on information that had transpired weeks or months before, as well as current level of depressive and anxiety symptoms that might affect reports of past suicidal ideation and plans,^45^ and thus recall bias and non-response bias could be introduced. Third, as is the case with other school-based surveys, SURF captured information only from students who attended school on the day of the survey, and this design could introduce selection bias due to attendance differences between our subgroups. For example, in the 2021 YRBSS,^5^ 23% of high school youth who identified as LGBQIA2S+ reported higher levels of bullying at school, and 14% reported not going to school because of safety concerns in the past 30 days, compared to 7% and 12% of their heterosexual counterparts, so it is possible that absenteeism rates in the LGBQIA2S+ may be disproportionately greater than for their heterosexual peers due to bullying and other negative school experiences that affect LGBQIA2S+ youth. Fourth, although these data were collected in 60 schools, all of these schools are in Massachusetts, most of them in urban areas, and this may limit generalizability to students in other states or in rural areas. Moreover, because of the Safe Schools Program, rates and percentages of mental health distress presented in this study might be lower than in other states. A fifth limitation is the small size of the GD only group, which might affect the survey’s internal validity. In this study, 53 students, which constitute 0.3% of the sample, identified as GD only, whereas 1,023 (4.8%) identified as having a SD+GD intersectional identity. Interestingly, the small size of the GD only subgroup is in line with a previous report that found that most adolescents who identified as GD also identified as SD.^22^ This small size is reflected in the uncertainty of the effect estimates, particularly for rare outcomes such as substance use. To address this, we performed a sensitivity analysis using the Fisher exact test to assess the effect of GD identity compared with HC and intersectional identity SD+GD on substance use. Results from the Fisher exact test and the unadjusted multivariate regression are very similar, increasing the certainty of the effect estimates (Table S5, available online). In addition, this survey was not only voluntary but also anonymous, thereby creating a limitation—we have no data on students who did not complete the survey, nor have we a way to identify them. Similarly, we do not have data on why parents opted out their students. This precluded our ability to probe any differences between students who participated in the survey and those who did not.

Massachusetts leads the nation in making substantial improvements in legal, social, and educational acceptance, providing safe and supportive environments for all, including LGBTQIA2+ students. Since 1993, the Safe Schools Program for LGBTQIA2+, a program sponsored by the Massachusetts Department of Elementary and Secondary Education (DESE) and the Massachusetts Commission on LGBTQ Youth, has been providing support to LGBTQIA2+ students and staff and helping schools implement Massachusetts laws such as the antibullying, gender identity, and student antidiscrimination laws.^48^ Despite the existence of these programs, high levels of negative mental health outcomes in this population persist, probably due to the complexity of mental health needs and the ability or inability of schools to adequately address these needs. Moreover, there are other factors that could have contributed to this persistence. Negative mental health outcomes among adolescents have been steadily increasing across all youth in the state, especially among female individuals and LGBTQIA2S+ students.^5^ Programs such as the Safe Schools Program follow a “one size fits all” approach and are not capable of taking into account specific identities that may be more disproportionately affected by mental health concerns. In addition, schools are also facing a shortage in mental health resources, especially post pandemic.^49^ This shortage leads schools to change or adapt the implementation of programs such as the Safe Schools Program, as there are not enough staff to follow through and check in after initial contact or to disseminate the Program at all. These issues result in students’ lack of awareness of the resources available to them, and decrease the likelihood of using these resources. Schools also continuously struggle to maintain a safe space or to base the program on the use of well-intentioned staff instead of trained staff, as other competing priorities may rely on the same resources.^50^

Our study is one of the first to characterize the risk profile of the understudied intersectional SD+GD identity, and highlights the significant disparities in mental health outcomes that exist among SD, GD, and intersectional SD+GD youth. This study has significant implications for provision of mental health care for adolescents with these intersectional identities, whose unique individual challenges are not addressed by the “one size fits all” model. These results also have important implications for pediatricians and mental health clinicians, drawing their attention to the alarming rates of depression, anxiety, and suicidality. Understanding these intersectional identities, the experiences and challenges that they face, and the needs that they have will inform the development of holistic, equitable, and inclusive programs and mental health services that take into account the complexities of having intersectional SD+GD identities. Such programs and services will also include providers that are trained in trauma-informed care and culturally sensitive practices and who are aware of these intersectionality and risk profile, with safe and affirming environments and systems that allow data collection, monitoring, and evaluation to ensure that youth needs are met. Future work should focus on examining factors responsible for the elevated risks in this population as well as protective factors that can be incorporated into prevention and intervention programs for this population.

## CRediT authorship contribution statement

**Gladys N. Pachas:** Writing – original draft, Visualization, Methodology, Formal analysis, Data curation, Conceptualization. **Harrison T. Reeder:** Writing – review & editing, Methodology, Formal analysis. **A. Eden Evins:** Writing – review & editing, Methodology, Conceptualization. **Kelly Casottana:** Writing – review & editing. **Marta Borrego Mahiques:** Writing – review & editing. **Alec Bodolay:** Writing – review & editing, Data curation. **Caroline A. Gray:** Writing – review & editing. **Kevin W. Potter:** Writing – review & editing, Data curation. **Alex S. Keuroghlian:** Writing – review & editing. **Randi M. Schuster:** Writing – review & editing, Supervision, Project administration, Methodology, Investigation, Funding acquisition, Conceptualization.
