# Supplementary material for: Substance Use, Suicidal Behavior, and Other Mental Health Outcomes Among Youth With Intersectional Sexual and Gender Diverse Identities
Source: JAACAP Open. 2025 Jan 9;3(4):947–58. doi: 10.1016/j.jaacop.2024.09.011 (PMC12684452; doi:10.1016/j.jaacop.2024.09.011)
Supplement: Supplemental Material [file mmc1.docx]

**SUPPLEMENTAL MATERIAL**

**Table S1. Distribution of Sexual Orientation and Gender Identity**

|  |  | Cisgender | Transgender | Non-Binary/other | I don't know/ I don't want to say | Total |
| --- | --- | --- | --- | --- | --- | --- |
| Heterosexual | n | 17,571 | 39 | 14 | 20 | 17,644 |
|  | %R | 99.6 | 0.2 | 0.1 | 0.1 | 100 |
|  | %C | 79.2 | 3.9 | 5.5 | 8.3 | 74.5 |
|  |  |  |  |  |  |  |
| Gay/Lesbian | n | 2,587 | 760 | 148 | 77 | 3,839 |
|  | %R | 72.4 | 21.3 | 4.1 | 2.2 | 100 |
|  | %C | 11.7 | 86.0 | 58.0 | 31.8 | 15.1 |
|  |  |  |  |  |  |  |
| Bisexual/other | n | 143 | 97 | 18 | 9 | 267 |
|  | %R | 53.6 | 36.3 | 6.7 | 3.4 | 100 |
|  | %C | 0.6 | 8.2 | 7.1 | 8.7 | 1.1 |
|  |  |  |  |  |  |  |
| I don't know/ I don't want to say | n | 1881 | 101 | 75 | 136 | 2,193 |
|  | %R | 85.8 | 4.6 | 3.4 | 6.2 | 100 |
|  | %C | 8.5 | 10.1 | 29.4 | 56.2 | 9.3 |
| Total | n | 22,182 | 997 | 255 | 242 | 23,676 |
|  | %R | 93.7 | 4. .2 | 1.1 | 1.0 | 100 |
|  | %C | 100 | 100 | 100 | 100 | 100 |

Note: %R (Row) = Distribution of sexual orientation by gender Identity; for example, 99.6% of heterosexual youth are cisgender, 0.22% are transgender, 0.1% are non-binary/other, and 0.1% don’t know/I don’t want to say.

%C (Column) = Distribution of gender identity by sexual orientation; for example, 79.1%. of cisgender youth are heterosexual, 12.3% are gay/lesbian, 5.5% are bisexual and 3.0% I don't know/ I don't want to say

**Table S2. Percentage of Adolescents with Sexual and Gender Diverse Identities by Middle and High school: Primary and Sensitivity Analysis**

|  | Primary Analysis: Undetermined responses excluded | Sensitivity Analysis: Undetermined + SD, GD | Sensitivity Analysis: Undetermined + HC |
| --- | --- | --- | --- |
| *Middle school* | % | % | % |
|  |  |  |  |
| HC | 85.4 | 72.7 | 85.2 |
| SD | 9.6 | 20.5 | 8.2 |
| GD | 0.3 | 0.4 | 0.3 |
| SD+GD | 4.8 | 6.4 | 6.4 |
|  |  |  |  |
|  |  |  |  |
| *High school* |  |  |  |
| HC | 80.8 | 75.0 | 80.8 |
| SD | 14.2 | 19.0 | 13.2 |
| GD | 0.2 | 0.3 | 0.2 |
| SD+GD | 4.8 | 5.8 | 5.8 |

Note: Undetermined responses= “not sure”, “questioning”, “I don’t want to say”; GD = Gender diverse; HC = hetero/cisgender; SD = Sexually diverse, SD+GD = intersectional sexual and gender diverse identities

**Table S3. Single and Compounding Effect of Sexual and Gender Identities on Mental Health Outcomes: Multivariate Regression (Unadjusted)**

| Measure | OR (95% CI) | | | | |
| --- | --- | --- | --- | --- | --- |
|  | GD only vs HC | SD only vs HC | SD + GD vs HC | SD + GD vs GD | SD + GD vs SD |
| Clinical Outcomes |  |  |  |  |  |
| Depression symptoms (PHQ4-D ≥3) | 2.3 (1.3 – 4.2)* | 3.8 (3.5 – 4.2)** | 6.4 (5.6 – 7.3)** | 2.8 (1.5-5.2)* | 1.7 (1.5-1.9)** |
| Anxiety symptoms (PHQ4-A ≥3) | 1.7 (0.9 – 3.0)^ns^ | 4.4 (4.0 – 4.8)** | 7.2 (6.3– 8.3)** | 4.4 (2.4-7.9)** | 1.7 (1.4-1.9)** |
| Psychotic experiences risk cut off (≤ 2) | 2.3 (1.2 – 4.1)* | 2.3 (2.1 – 2.5 )** | 4.6 (4.0 – 5.2)** | 2.0 (1.1-3.7)* | 2.0 (1.7-2.3)** |
| Suicidal thoughts & behaviors (past year) |  |  |  |  |  |
| Thoughts | 3.5 (1.9 – 6.3)** | 5.2 (4.7 – 5.6)** | 10.0 (8.8 – 11.5)** | 2.9 (1.6-5.3)* | 2.0 (1.7-2.3)** |
| Plan | 3.6 (1.9 – 6.7)** | 4.7 (4.3 – 5.2)** | 8.3 (7.2 – 9.5)** | 1.8 (1.5-2.0)** | 1.8 (1.5-2.0)** |
| Attempt | 6.0^a^ (2.4–15.1)** | 5.6 (4.7 – 6.6 )** | 10.0^a^ (8.1– 12.3)** | 1.7^a^ (0.7-4.3)^ns^ | 1.8^a^ (1.4-2.2)** |
| Non-suicidal self-harm | 2.7 (1.3 – 5.6 )* | 5.1 (4.6 – 5.6)** | 10.0 (8.8 – 11.5)** | 3.7 (1.8-7.6)** | 2.0 (1.7-2.3)** |
| Substance Use (past month) |  |  |  |  |  |
| Alcohol (≥ weekly use) | 4.5 (1.8 – 11.4)* | 1.3 (1.0 – 1.7)* | 1.8 (1.3 – 2.5)** | 0.40 (0.2-1.1)^ns^ | 1.4 (0.9-2.0)^ns^ |
| Cannabis (≥ weekly use) | 3.7 (1.5 – 9.4)* | 2.7 (2.3 – 3.2)** | 2.7 (2.1 – 3.5)** | 0.7 (0.3-1.9)^ns^ | 1.0 (0.8-1.3)^ns^ |
| Smoking tobacco (≥ weekly use) | 41.1^a^ (15.6-108.0)** | 2.9 (1.6 - 4.8)** | 12.0^a^ (7.5 – 19.2)** | 0.7^a^ (0.3-1.6)^ns^ | 4.3^a^ (2.4-7.7)** |
| Vaped nicotine (≥ weekly use) | 1.9 (0.6 – 6.1)^ns^ | 2.4 (2.0 - 2.8)** | 2.2 (0.8 - 1.4)** | 1.7 (0.7-4.3)^ns^ | 0.9 (0.7-1.3)^ns^ |
|  | Δ score (95% CI) | | | | |
|  | GD only vs HC | SD only vs HC | SD + GD vs HC | SD + GD vs GD | SD + GD vs SD |
| ERS (total score) | 10.5 (5.2–15.8)** | 18.6 (17.8–19.3)** | 27.3 (26.0–28.5)** | 16.8 (11.4-22.2)** | 8.7 (7.3-10.1)** |

Note: P values have been corrected for multiple comparisons with Benjamini-Hochberg. a Due to small sample size, comparisons including the GD group should be considered as suggestive findings for smoking tobacco and suicidal attempts. APSS = Adolescent Psychotic-like Symptom Screener; ERS = Emotional Reactivity Scale total score; GD = Gender diverse; HC = hetero/cisgender; PHQ-A = Patient Health Questionnaire-Anxiety subscale; PHQ-D = Patient Health Questionnaire-Depression subscale; SD = Sexually diverse, SD+GD = intersectional sexual and gender diverse identities.

*pvalue <0.05; ** pvalue <0.001; ns = non-significant

Supplement 1

Sensitivity Analysis

Since 9% of the responses to the SD or GD questions were classified as undetermined (not sure, questioning, I don’t want to say) and excluded from the main analysis, a sensitivity analysis was performed to examine the single and synergistic effects of SD and GD on mental health outcomes when responses from the undetermined group were added to the corresponding SD or GD groups or the HC group, resulting 2 models:

**Model 1SA**: HC, GD + corresponding undetermined (GD1), SD + corresponding undetermined (SD1), SD1+GD1

When added to the SD or GD groups (Table S4a), effect sizes and significance remained for all outcomes except for alcohol and cannabis use. For alcohol, GD only vs HC was no longer significant (OR=3.4, 95%CI 1.1-9.9 vs. OR=2.3, 95%CI 0.8-6.5, p=0.108) and SD+GD vs SD is now significant (OR=1.5, 95%CI 1.0-2.2 vs. OR=1.9, 95%CI 1.3-2.7, p<0.001). For cannabis, GD only vs HC is no longer significant (OR=2.9, 95%CI 1.0-8.4 vs. OR=2.5, 95%CI 1.0-6.3, p=0.06).

**Model 2SA:** HC + undetermined, GD, SD, SD+GD

When added to the HC group (Table S4b), effect sizes and significance remained, except for depression and self-harm. For depression, the effect of SD+GD when compared to GD is no longer significant (OR=2.4, 98%CI 1.3-4.5 vs OR=1.3, 95%CI 1.0-1.8, p=0.07). Similarly, for self-harm, the effect of SD+GD when compared to GD is no longer significant (OR=3.0, 95%CI 1.4-6.3 vs. OR=1.3, 95%CI 1.0-1.8, p=0.07)

Table S4a. Single and Synergistic Effect of Sexual and Gender Identities on Mental Health Outcomes Adjusted (by sex, age): Model 1, undetermined responses added to the SD and GD group

| Measure | OR (95% CI) | | | | |
| --- | --- | --- | --- | --- | --- |
|  | GD only vs HC | SD only vs HC | SD + GD vs HC | SD + GD vs GD | SD + GD vs SD |
| Clinical Outcomes |  |  |  |  |  |
| Depression symptoms (PHQ4-D ≥3) | 2.5 (1.5-4.2)* | 2.5 (2.3 – 2.7)** | 4.9 (4.3-5.5)** | 2.0 (1.2-3.3)* | 1.9 (1.7-2.2)** |
| Anxiety symptoms (PHQ4-A ≥3) | 2.2 (1.3-3.6)* | 2.5 (2.4 – 2.7)** | 4.7 (4.2-5.3)** | 2.2 (1.3-3.7)* | 1.9 (1.6-2.1)** |
| Psychotic experiences risk cut off (≤ 2) | 2.3 (1.4-3.9)* | 2.0 (1.8 – 2.1)** | 4.2 (3.7-4.7)** | 1.8 (1.1-3.1)* | 2.1 (1.9-2.4)** |
| Suicidal thoughts & behaviors (past year) |  |  |  |  |  |
| Thoughts | 3.3 (1.9-5.5)** | 3.1 (2.9-3.4)** | 7.2 (6.4-8.1)** | 2.2 (1.3-3.8)* | 2.3 (2.0-2.6)** |
| Plan | 3.0 (1.7-5.3)** | 3.2 (2.9-3.4)** | 6.5 (5.7-7.3)** | 2.2 (1.2-3.9)* | 2.1 (1.8-2.4)** |
| Attempt | 4.5^a^ (1.8-11.2)* | 3.5 (2.9-4.1)** | 7.9^a^ (6.5-9.7)** | 1.8^a^ (0.7-4.5)^ns^ | 2.3^a^ (1.9-2.8)** |
| Non-suicidal self-harm | 2.9 (1.6-5.5 )* | 3.1 (2.9-3.4)** | 7.2 (6.4-8.2)** | 2.5 (1.3-4.6)* | 2.3 (2.0-2.6)** |
| Substance Use (past month) |  |  |  |  |  |
| Alcohol (≥ weekly use) | 2.3^b^ (0.8-6.5)^ns^ | 0.9 (0.8-1.2)^ns^ | 1.8 (1.3-2.4)** | 0.7 (0.3-2.2)^ns^ | 1.9^c^ (1.3-2.7)** |
| Cannabis (≥ weekly use) | 2.5^b^ (1.0-6.3)^ns^ | 2.1 (1.8-2.5)** | 2.7 (2.1-3.5)** | 1.1 (0.4-2.8)^ns^ | 1.3 (1.0-1.7)^ns^ |
| Smoking tobacco (≥ weekly use) | 23.0^a^ (8.2-64.2)** | 2.6 (1.5-4.5)** | 13.8^a^ (8.5-22.3)** | 0.6^a^ (0.2-1.7)^ns^ | 5.2^a^ (3.0-9.1)** |
| Vaped nicotine (≥ weekly use) | 1.9 (0.7-5.1)^ns^ | 1.6 (1.3-1.8)** | 1.8 (1.4-2.3)** | 0.9 (0.3-2.5)^ns^ | 1.2 (0.9-1.5)^ns^ |
|  | Δ score (95% CI) | | | | |
|  | GD only vs HC | SD only vs HC | SD + GD vs HC | SD + GD vs GD | SD + GD vs SD |
| ERS (total score) | 10.3 (5.8-14.7)** | 11.0 (10.4–11.7 )** | 20.8 (19.8-21.9)** | 10.6 (6.0-15.1)** | 9.8 (8.6-10.9)** |

Note: ^a^ Due to small sample size, comparisons including the GD group should be considered as suggestive findings for smoking tobacco and suicidal attempts; ^b^ When compared to the main analysis, results no longer significant; ^c^ When compared to main analysis, results are now significant. P values have been corrected for multiple comparisons with Benjamini-Hochberg. a Due to small sample size, comparisons including the GD group should be considered as suggestive findings for smoking tobacco and suicidal attempts. APSS = Adolescent Psychotic-like Symptom Screener; ERS = Emotional Reactivity Scale total score; GD = Gender diverse; HC = hetero/cisgender; PHQ-A = Patient Health Questionnaire-Anxiety subscale; PHQ-D = Patient Health Questionnaire-Depression subscale; SD = Sexually diverse, SD+GD = intersectional sexual and gender diverse identities.

*pvalue <0.05; ** pvalue <0.001; ns = non-significant

Table S4b. Single and Synergistic Effect of Sexual and Gender Identities on Mental Health Outcomes Adjusted (by sex, age): Model 2, undetermined responses added to the HC group

| Measure | OR (95% CI) | | | | |
| --- | --- | --- | --- | --- | --- |
|  | GD only vs HC | SD only vs HC | SD + GD vs HC | SD + GD vs GD | SD + GD vs SD |
| Clinical Outcomes |  |  |  |  |  |
| Depression symptoms (PHQ4-D ≥3) | 3.9 (3.0-5.1)** | 3.0 (2.8 – 3.3)** | 5.1 (4.5-5.9)** | 1.3^b^ (1.0-1.8)^ns^ | 1.7 (1.5-2.0)** |
| Anxiety symptoms (PHQ4-A ≥3) | 3.0 (2.3-3.9)** | 3.0 (2.8 – 3.3)** | 5.2 (4.6-6.0)** | 1.8 (1.3-2.4)** | 1.8 (1.5-2.0)** |
| Psychotic experiences (APSS ≥ 2) | 3.1 (2.4-4.1)** | 2.4 (2.1 – 2.6)** | 4.4 (3.8-5.0)** | 1.4 (1.0-1.9)* | 1.9 (1.6-2.2)** |
| Suicidal Thoughts and Behaviors (past year) |  |  |  |  |  |
| Thoughts | 6.4 (4.0-6.8)** | 4.2 (3.8-4.6)** | 8.2 (7.2-9.4)** | 1.6 (1.2-2.1)* | 2.0 (1.7-2.3)** |
| Plan | 4.6 (3.4-6.1)** | 4.0 (3.6-4.4)** | 7.0 (6.1-8.0)** | 1.5 (1.1-2.1)* | 1.8 (1.5-2.0)** |
| Attempt | 7.1^a^ (4.7-10.7)** | 4.6 (3.9-5.5)** | 8.1^a^ (6.5-9.9)** | 1.1^a^ (0.7-1.8)^ns^ | 1.7^a^ (1.4-2.2)** |
| Non-suicidal self-harm | 5.6 (4.2-7.5)** | 3.9 (3.5-4.3)** | 7.5 (6.5-8.6)** | 1.3^b^ (1.0-1.8)^ns^ | 1.9 (1.7-2.3)** |
| Substance Use (past month) |  |  |  |  |  |
| Alcohol (≥ weekly use) | 3.4 (1.9-6.2)* | 1.2 (1.0 – 1.6)^ns^ | 1.8 (1.3-2.6)* | 0.5 (0.3-1.1)^ns^ | 1.5 (1.0-2.2)^ns^ |
| Cannabis (≥ weekly use) | 2.6 (1.4-4.8)* | 2.8 (2.3 – 3.3)** | 3.1 (2.3-4.0)** | 1.2 (0.6-2.3)^ns^ | 1.1 (0.8-1.5)^ns^ |
| Smoking tobacco (≥ weekly use) | 21.9^a^ (10.2-47.1)** | 3.7 (2.1 – 6.4)** | 15.9^a^ (9.6-26.5)** | 0.7^a^ (0.3-1.6)^ns^ | 4.4^a^ (2.4-8.0)** |
| Vaped nicotine (≥ weekly use) | 1.7 (0.9-3.3)^ns^ | 2.0 (1.7 – 2.4)** | 2.0 (1.5-2.7)** | 1.2 (0.6-2.3)^ns^ | 1.0 (0.8-1.4)^ns^ |
|  | Δ score (95% CI) | | | | |
|  | GD only vs HC | SD only vs HC | SD + GD vs HC | SD + GD vs GD | SD + GD vs SD |
| ERS (total score) | 16.4 (13.8-18.9)** | 14.3 (13.5–15.1 )** | 22.6 (21.4-23.9)** | 6.3 (3.5-9.0)** | 8.3 (7.0-9.7)** |

Note: ^a^ Due to small sample size, comparisons including the GD group should be considered as suggestive findings for smoking tobacco and suicidal attempts; ^b^ When compared to the main analysis, results no longer significant; ^c^ When compared to main analysis, results are now significant. P values have been corrected for multiple comparisons with Benjamini-Hochberg. a Due to small sample size, comparisons including the GD group should be considered as suggestive findings for smoking tobacco and suicidal attempts. APSS = Adolescent Psychotic-like Symptom Screener; ERS = Emotional Reactivity Scale total score; GD = Gender diverse; HC = hetero/cisgender; PHQ-A = Patient Health Questionnaire-Anxiety subscale; PHQ-D = Patient Health Questionnaire-Depression subscale; SD = Sexually diverse, SD+GD = intersectional sexual and gender diverse identities.

*pvalue <0.05; ** pvalue <0.001; ns = non-significant

**Table S5. Single and Compounding Effect of Sexual and Gender Identities on Mental Health Outcomes: unadjusted odds ratios and confidence intervals based on Fisher’s exact test**

| Measure | OR (95% CI) | | | | |
| --- | --- | --- | --- | --- | --- |
|  | GD only vs HC | SD only vs HC | SD + GD vs HC | SD + GD vs GD | SD + GD vs SD |
| Clinical Outcomes |  |  |  |  |  |
| Depression symptoms (PHQ4-D ≥3) | 2.3 (1.2 - 4.3)* | 3.8 (3.5 - 4.1)** | 6.4 (5.6 - 7.3)** | 2.8 (1.5 - 5.5)* | 1.7 (1.5 - 1.9)** |
| Anxiety symptoms (PHQ4-A ≥3) | 1.7 (0.9 - 3.1)^ns^ | 4.3 (4.0 - 4.7)** | 7.2 (6.2 - 8.2)** | 4.3 (2.3 - 8.3)** | 1.6 (1.4 - 1.9)** |
| Psychotic experiences risk cut off (≤ 2) | 2.3 (1.2 - 4.3)* | 2.2 (2.0 - 2.5)** | 4.5 (3.9 - 5.1)** | 1.9 (1.0 - 3.9)* | 2.0 (1.7 - 2.3)** |
| Suicidal thoughts & behaviors (past year) |  |  |  |  |  |
| Thoughts | 3.5 (1.8 - 6.5)** | 5.1 (4.7 - 5.6)** | 9.9 (8.6 - 11.3)** | 2.8 (1.5 - 5.5)* | 1.9 (1.7 - 2.2)** |
| Plan | 3.6 (1.8 - 6.9)** | 4.7 (4.3 - 5.2)** | 8.2 (7.1 - 9.4)** | 2.3 (1.2 - 4.7)* | 1.7 (1.5 - 2.0)** |
| Attempt | 6.0 (1.8 - 15.1)* | 5.5 (4.6 - 6.6)** | 9.9 (8.0 - 12.2)** | 1.6 (0.6 - 5.4)^ns^ | 1.8 (1.4 - 2.2)** |
| Non-suicidal self-harm | 2.8 (1.2 - 5.7)* | 5.0 (4.5 - 5.6)** | 9.9 (8.6 - 11.4)** | 3.6 (1.7 - 8.5)** | 2.0 (1.7 - 2.3)** |
| Substance Use (past month) |  |  |  |  |  |
| Alcohol (≥ weekly use) | 4.5 (1.4 - 11.4)* | 1.3 (1.0 - 1.7)* | 1.8 (1.3 - 2.5)** | 0.4 (0.1 - 1.4)^ns^ | 1.4 (0.9 - 2.1)^ns^ |
| Cannabis (≥ weekly use) | 3.7 (1.1 - 9.3)* | 2.7 (2.2 - 3.2)** | 2.7 (2.1 - 3.5)** | 0.7 (0.3 - 2.4)^ns^ | 1.0 (0.7 - 1.3)^ns^ |
| Smoking tobacco (≥ weekly use) | 41.4 (12.3-110.4)** | 2.8 (1.5-4.9)** | 12.0 (7.3-19.7)** | 0.3 (0.1-1.0)* | 4.3 (2.3 - 8.1)** |
| Vaped nicotine (≥ weekly use) | 1.9 (0.4 - 6.0)^ns^ | 2.4 (2.0 - 2.8)** | 2.2 (1.7 - 2.9)** | 1.2 (0.4 - 6.0)^ns^ | 0.9 (0.7 - 1.3)^ns^ |
|  | Δ score (95% CI) | | | | |
|  | GD only vs HC | SD only vs HC | SD + GD vs HC | SD + GD vs GD | SD + GD vs SD |
| ERS (total score) | 10.5 (5.6 - 15.3)** | 18.6 (17.8-19.3)** | 27.3 (26.1-28.4)** | 16.8 (10.4 - 23.2)** | 8.7 (7.1 - 10.3)** |

Note: We estimated unadjusted odds ratios and confidence intervals based on Fisher’s exact test. For each outcome we reported unadjusted odds ratios for the intersectional SD+GD identity versus HC, GD only and SD only identities. This highlighted the impact on mental health outcomes of intersectional identity relative to SD identity alone or GD identity alone and HC. We used a similar approach for the continuous outcome of ERS total score, considering unadjusted linear regression models. APSS = Adolescent Psychotic-like Symptom Screener; ERS = Emotional Reactivity Scale total score; GD = Gender diverse; HC = hetero/cisgender; PHQ-A = Patient Health Questionnaire-Anxiety subscale; PHQ-D = Patient Health Questionnaire-Depression subscale; SD = Sexually diverse, SD+GD = intersectional sexual and gender diverse identities.

*pvalue <0.05; ** pvalue <0.001; ns = non-significant

Supplement 2

Mental health resources included in every survey

**This request is NOT monitored in real-time. There are some resources below if you need more immediate help. You may want to take a picture of these so you can look at them later.**

*If you are concerned that you or someone else may be at risk for suicide:*

- Please call: 911 or 988 (Suicide and Crisis Lifeline)
- Youth Peer-to-Peer Line: Call (877) 968-8491 or Text 839-863
- Kiva Center Warm Line: (508) 688-5898

*If you are concerned about your substance use:*

- MA Substance Use Helpline: Call or text (800) 327-5050 or go to helplinema.org
- This is Quitting: Text “VapeFreeMass” to 88709 or go to truthinitiative.org

*If you are concerned about other aspects of your mental health:*

- MA Behavioral Health Help Line: Call or text 833-773-2445 or go to masshelpline.com
- Crisis Text Line: Text HOME to 741-741
